# Methodological issues on evaluating agreement between two detection methods by Cohen’s kappa analysis

**DOI:** 10.1186/s13071-022-05402-8

**Published:** 2022-07-29

**Authors:** Ming Li, Tianfei Yu

**Affiliations:** 1 College of Computer and Control Engineering, Qiqihar University, Qiqihar, 161006 China; 2 College of Life Science and Agriculture Forestry, Qiqihar University, Qiqihar, 161006 China

**Keywords:** CSP ELISA, COX-I PCR, Plasmodium, Mosquitoes, Cohen’s kappa

## Abstract

We read with great interest the article by Hendershot et al. (Parasit Vectors 14:473, 2021). The authors compared a PCR method for detecting *Plasmodium vivax*’s mitochondrial (mt) cytochrome oxidase I (*COX-I*) gene with the current “gold standard” circumsporozoite (CSP) ELISA for detecting circumsporozoite protein for identification of different life stages of *Plasmodium vivax* during development within *Anopheles arabiensis*. We found that Cohen’s kappa value for measuring the agreement between mt COX-I PCR and CSP ELISA was questionable. In addition, we recommend a more appropriate statistical method in this article.

In short, any scientific conclusion requires support by the reasonable application of methodological and statistical methods.

**To the Editor,**

We read with interest the article entitled: “A comparison of PCR and ELISA methods to detect different stages of *Plasmodium vivax* in *Anopheles arabiensis*,” which was published in *Parasites and Vectors* on 15 September 2021 [1]. The authors compared a PCR method for detecting *Plasmodium vivax*’s mitochondrial (mt) cytochrome oxidase I (*COX-I*) gene with the current “gold-standard” circumsporozoite (CSP) ELISA for detecting circumsporozoite protein for identification of different life stages of *Plasmodium vivax* during development within *Anopheles arabiensis*. They evaluated the agreement between the results of the mt COX-I PCR and the CSP ELISA by using Cohen’s kappa.

Generally, Cohen’s kappa [2] is calculated as follows:

1 $${k}_{C}=\frac{\sum_{j=1}^{n}{u}_{jj}\left(i{i}^{^{\prime}}\right)-\sum_{j=1}^{n}{p}_{ij}{p}_{{i}^{^{\prime}}j}}{1-\sum_{j=1}^{n}{p}_{ij}{p}_{{i}^{^{\prime}}j}}$$

The value of ${u}_{jj}\left(i{i}^{^{\prime}}\right)$ is the proportion of objects put in the same category j by both raters $i$ and ${i}^{^{\prime}}$. The value of ${p}_{ij}$ is the proportion of objects that rater $i$ assigned to category $j$, and $k$ is the number of raters. Cohen suggested the *k* value be interpreted as follows: *k* ≤ 0 as indicating no agreement and 0.01–0.20 as none to slight, 0.21–0.40 as fair, 0.41–0.60 as moderate, 0.61–0.80 as substantial, and 0.81–1.00 as almost perfect agreement [2]. According to the author’s description, according to Cohen’s interpretation for *k* value, the agreement between mt COX-I PCR and CSP ELISA was “fair” for mosquitoes bisected at 9–15 dpi in the head and thorax (*κ* = 0.312).

Although this article has provided valuable information, some substantial points that may lead to misinterpretation of the results need to be clarified. Unlike the authors, for 9–15 dpi, we calculated the agreement between mt COX-I PCR and CSP ELISA with SPSS 18 statistical package (SPSS 18 Inc., Chicago, IL, USA) software. The kappa values in head and thorax and abdomen samples were 0.299 and 0.304, respectively. Furthermore, a simple sum of the data was performed, and the kappa value obtained was 0.302 (Table 1). Each of the three kappa values was different from the authors’ kappa value of 0.312. We would be grateful if the authors could explain their results in detail and clarify the misunderstanding.

**Table 1:** Kappa values for calculating agreement between COX-I PCR and CSP ELISA for 9–15 dpi

Body part	CSP ELISA				*k* value
COX-I PCR					
Head and thorax		Positive	Negative	Total	*k* = 0.312 (k* = 0.299)
	Positive	84	101	185	
	Negative	5	78	83	
	Total	89	179	268	
Abdomen		Positive	Negative	Total	k* = 0.304
	Positive	79	97	176	
	Negative	6	83	89	
	Total	85	180	265	
Head and thorax + abdomen		Positive	Negative	Total	k* = 0.302
	Positive	163 (84 + 79)	198 (84 + 79)	361	
	Negative	11 (5 + 6)	161 (78 + 83)	172	
	Total	174	359	533	

The data are cited from the article published by Hendershot et al. [1] and underwent modification

*k* in the table is the kappa value calculated by Hendershot et al., and k* is the kappa value calculated by us.

Furthermore, McHugh [4] provided a more logical interpretation of *k* value: 0–0.20 = no agreement, 0.21–0.39 = minimal agreement, 0.40–0.59 = weak agreement, 0.60–0.79 = moderate agreement, 0.80–0.90 = strong agreement, and 0.91–1.00 = almost perfect agreement. McHugh stated that: “For percent agreement, 61% agreement can immediately be seen as problematic. Almost 40% of the data in the data set represent faulty data. In healthcare research, this could lead to recommendations for changing practice based on faulty evidence. For a clinical laboratory, having 40% of the sample evaluations being wrong would be an extremely serious quality problem. This is the reason that many texts recommend 80% agreement as the minimum acceptable interrater agreement. Given the reduction from percent agreement that is typical in kappa results, some lowering of standards from percent agreement appears logical. However, accepting 0.40 to 0.60 as ‘moderate’ may imply the lowest value (0.40) is adequate agreement.” Therefore, we also recommend the authors use McHugh’s interpretation to replace Cohen’s interpretation to analyze the kappa values. In a word, any scientific conclusion needs to be supported by the reasonable application of methodological and statistical methods. Using appropriate statistical methods can improve the scientific nature of research results.

## Data Availability

Not applicable.
